# Association of serum insulin-like growth factor-1 and adrenocorticotropic hormone therapeutic response in patients with infantile epileptic spasms syndrome

**DOI:** 10.3389/fphar.2025.1599641

**Published:** 2025-04-30

**Authors:** Ka-Na Lin, Feng Han, Ying-Yan Wang, Wei Zhao, Ji-Wen Wang, Hao Li, Yun-Qing Zhou

**Affiliations:** ^1^ Department of Neurology, Shanghai Children’s Medical Center, Shanghai Jiao Tong University School of Medicine, Shanghai, China; ^2^ Clinical Research Ward, Clinical Research Center, Shanghai Children’s Medical Center, Shanghai Jiao Tong University School of Medicine, Shanghai, China; ^3^ Department of Pharmacy, Shanghai Children’s Medical Center, Shanghai Jiao Tong University School of Medicine, Shanghai, China; ^4^ Department of Neurology, Hainan Branch, Shanghai Children’s Medical Center, Shanghai Jiao Tong University School of Medicine, Sanya, Hainan, China

**Keywords:** insulin-like growth factor-1, insulin-like growth factor-binding protein-3, adrenocorticotropic hormone, infantile epileptic spasms syndrome, video-electroencephalogram, rare disease

## Abstract

**Background:**

Infantile epileptic spasm syndrome (IESS), a rare age-specific epileptic encephalopathy, exhibits limited therapeutic efficacy, with approximately 50% of patients showing resistance to adrenocorticotropic hormone (ACTH) monotherapy. Herein, we investigated the association between serum insulin-like growth factor-1 (IGF-1), insulin-like growth factor-binding protein-3 (IGFBP-3), their ratio, and short-term ACTH therapeutic response in IESS, alongside their correlation with video-electroencephalogram (VEEG) characteristics.

**Methods:**

This retrospective study included IESS patients who received ACTH treatment at Shanghai Children’s Medical Center from July 2021 to November 2024. Clinical data, including serum IGF-1, IGFBP-3 levels, VEEG findings, and short-term treatment responses, were collected. Before ACTH therapy, we classified patients into hypsarrhythmia and non-hypsarrhythmia groups based on VEEG findings. The hypsarrhythmia cohort was further subdivided into ACTH responders and non-responders. Statistical analyses employed independent t-tests, Mann-Whitney U tests, chi-square tests, and Spearman’s rank correlation.

**Results:**

A total of 21 patients (14 hypsarrhythmia, 7 non-hypsarrhythmia) were enrolled. The hypsarrhythmia population exhibited significantly lower serum IGF-1 levels and IGF-1/IGFBP-3 ratios (p < 0.05) compared to the non-hypsarrhythmia population. Within the hypsarrhythmia population, responders (n = 9) showed higher IGF-1, IGFBP-3 levels, and IGF-1/IGFBP-3 ratios than non-responders (n = 5) before ACTH treatment (p < 0.05). Post-ACTH treatment, serum IGF-1 and IGFBP-3 levels increased in all patients, with greater elevation observed in responders.

**Conclusion:**

Our findings demonstrate that serum IGF-1, IGFBP-3 levels, and their ratio correlate with both hypsarrhythmia severity and short-term ACTH response in IESS patients. These biomarkers may help guide personalized treatment decisions.

## 1 Introduction

Infantile epileptic spasms syndrome (IESS), formerly termed infantile spasms (IS), is a rare age-specific epileptic encephalopathy that includes patients who do not fully meet the criteria for West syndrome (Zuberi et al., 2022; Pavone et al., 2020). Recent studies indicate an incidence of 30 per 100,000 live births, with a slight male predominance (Symonds et al., 2021). Although peak onset occurs between 6 and 7 months of age, diagnostic criteria allow for 3–24 months age range (Velisek and Veliskova, 2020). IESS severely impacts neurodevelopment and imposes significant socioeconomic burdens (Riikonen, 2020). Current therapeutic approaches including adrenocorticotropic hormone (ACTH), prednisolone, vigabatrin, ketogenic diet, or combination therapy (Dozieres-Puyravel et al., 2024), exhibit variable efficacy, with approximately 50% of patients showing resistance to ACTH monotherapy (Jia et al., 2013; Hussain, 2018; Wang et al., 2022; He et al., 2024). Notably, vigabatrin shows superior efficacy versus ACTH or corticosteroids for patients with structural etiologies like tuberous sclerosis complex (Overwater et al., 2015). The development of biomarkers to predict the therapeutic outcomes of IESS is critically important.

Insulin-like growth factor-1 (IGF-1) is a single-chain polypeptide hormone primarily synthesized in the liver, with minor production in the brain (Dyer et al., 2016). Most cerebral IGF-1 originates from peripheral circulation. This hormone serves as an important anti-apoptotic factor, contributing to neurodevelopment and neuroprotection through its anti-inflammatory effects. (Corvin et al., 2012; Lin et al., 2022). Lower cerebrospinal fluid (CSF) IGF-1 levels in IESS patients associate with both poorer treatment responses and greater cognitive decline (Riikonen et al., 2010). In resected brain tissue from IESS patients with perinatal stroke, IGF-1 expression shows significant downregulation, consistent with findings in animal models (Ballester-Rosado et al., 2022). In animal models, IGF-1 administration effectively eliminates both spasms and hypsarrhythmia, indicating its potential utility as both a therapeutic agent and disease severity biomarker (Ballester-Rosado et al., 2022; Yamada et al., 2014). Hypsarrhythmia correlates with clinical severity markers, including higher spasm frequency and neurodevelopmental regression, and primarily occurs during non-REM sleep, though not all patients exhibit this pattern (Mytinger et al., 2015; Gaily et al., 2001). The resolution of hypsarrhythmia following ACTH therapy predicts favorable outcomes, demonstrating the connection between electroencephalogram (EEG) improvements and treatment efficacy (Yamada et al., 2014). These findings establish important interrelationships between IGF-1 levels, video-electroencephalogram (VEEG) patterns, and ACTH responsiveness, highlighting IGF-1’s dual potential as both a diagnostic biomarker and therapeutic target in IESS.

Although previous studies (Ballester-Rosado et al., 2022; Mytinger et al., 2015; Riikonen, 2023) have suggested potential associations among IGF-1, VEEG, and ACTH therapeutic outcomes, the relationship between IGF-1 and VEEG and their synergistic effects on ACTH treatment prognosis remains unclear. Furthermore, current research on IGF-1 in IESS has focused predominantly on the central nervous system (e.g., CSF and brain tissue), with limited attention given to peripheral blood IGF-1 concentrations. In peripheral circulation, IGFBP-3 forms complexes with IGF-1 and plays a critical role in regulating free IGF-1 levels in blood and sustaining its biological activity. Thus, the IGF-1/IGFBP-3 ratio serves as a reliable estimator of bioactive and free IGF-1 (Juul et al., 1995). In this study, we collected clinical data from IESS patients via real-world research to analyze associations among serum IGF-1, IGFBP-3 levels, their ratio, and VEEG findings. Building on these observations, we investigated their association with ACTH treatment response by evaluating both epileptic spasm changes and EEG improvements after 2 weeks of therapy (Frost et al., 1978; Kellaway et al., 1979). Additionally, we monitored post-treatment IGF-1 and IGFBP-3 levels and their ratio to provide evidence for timely clinical diagnosis, treatment optimization, and enhanced therapy management.

## 2 Materials and methods

### 2.1 Study design and population

This is a retrospective study involving patients with IESS admitted to Shanghai Children’s Medical Center, Shanghai Jiao Tong University School of Medicine between July 2021 and November 2024. The diagnostic criteria for IESS follow the 2022 International League Against Epilepsy (ILAE) classification of epilepsy syndromes (Zuberi et al., 2022). We established an electronic database for enrolled IESS patients who received ACTH treatment and met the eligibility criteria: (1) age between 1 and 24 months; (2) underwent ACTH treatment during hospitalization; Exclusion criteria included: (1) loss of serum IGF-1 or IGFBP-3 measurements before ACTH treatment; (2) presence of complications that may affect IGF-1 test results, such as diabetes, current infection, hypothyroidism, liver disease, tumor disease or surgery; (3) incomplete ACTH treatment plan; (4) prior use of prednisone before ACTH therapy. The study protocol, informed consent document, and research materials were approved by the Ethics Committee of Shanghai Children’s Medical Center (approval number: SCMCIRB-K2023015-1).

### 2.2 ACTH pharmacotherapeutic regimen

The treatment protocol was designed with reference to the ACTH and prednisone sequential therapy recommended in the 2012 guidelines from the American Academy of Neurology and Child Neurology Society (Go et al., 2012), while incorporating our center’s clinical experience. The regimen is as follows: Based on age and weight, the initial ACTH dose was administered intramuscularly at 12.5U or 25U, 1–2 times daily. During treatment, ACTH dosage was adjusted using dose escalation/tapering strategies according to therapeutic response and adverse effects. Following ACTH completion, patients transitioned to oral prednisone acetate at 2 mg/(kg·d). Steroid tapering commenced after 2 weeks, with total treatment duration spanning 3–6 months. ACTH specification was 25U/tube (manufactured by Shanghai First Biochemical & Pharmaceutical Co., Ltd., Shanghai, China, with national drug approval number H31022101).

### 2.3 Data collection

Clinical data collection included: General information: age, gender, height, weight, birth history, past medical history, and developmental milestones; Auxiliary examinations: serum IGF-1 and IGFBP-3 levels, video electroencephalogram (VEEG), cranial MRI results; IESS-related data: age at onset, seizure frequency, interval between seizure onset and ACTH treatment initiation, use of standard first-line treatments, and second-line therapeutic agents.

Throughout the ACTH treatment protocol, two experienced neurologists conducted thorough clinical evaluations. Patients were categorized into either non-hypsarrhythmia population or hypsarrhythmia population based on VEEG findings. Further, within the hypsarrhythmia subgroup, patients were classified into non-response population or a response population based on short-term treatment outcomes. Throughout the disease course, the basic clinical data and treatment strategies for both groups were carefully evaluated and reviewed.

### 2.4 VEEG monitoring

VEEG monitoring was performed using a 32-channel Natus Medical Incorporated system. Electrode placement strictly adhered to the international 10–20 system, with recordings lasting ≥2 h to capture at least one complete wake-sleep cycle. Monitoring was conducted both pre-ACTH treatment and on day 14 of therapy. EEG interpretation followed diagnostic criteria from Clinical Electroencephalography and the 2017 ILAE guidelines for IESS (Scheffer et al., 2017).

### 2.5 Criteria for determining short-term response

Short-term response was defined by complete cessation of epileptic spasms and VEEG improvement after 2 weeks of ACTH treatment, primarily evaluated through parental reports and pre-/post-treatment VEEG comparisons (Takacs et al., 2023). Notably, discrepancies may arise between parental observations and objective EEG findings due to potential underestimation of epileptic spasms frequency or persistence by caregivers (Frost et al., 1978; Kellaway et al., 1979).

#### 2.5.1 Epileptic spasms (ES) assessment

At the conclusion of the therapeutic regimen, we evaluated the occurrence of ES after ACTH clinical treatment response. The criteria for judgment were as follows: (1) “complete response” (CR): complete cessation of spasms; (2) “partial response” (PR): a reduction in seizure frequency by >50%; (3) “no response” (NR): the frequency of seizures had been reduced by <50% or had not been reduced at all (Wan et al., 2021). Patients achieving CR were categorized as the ES-free group, whereas those with PR and NR were classified as the ES-unceased group.

#### 2.5.2 VEEG improvement assessment

The VEEG resolution should include suppression of both spasms and hypsarrhythmia. The BASED scoring system was employed for quantitative EEG evaluation (Mytinger et al., 2021). During sleep phases with maximal abnormal discharge frequency, a 5-min epoch was selected. Patient EEG data were scored as follows: five points: ≥3 spike foci and ≥50% channels showing abnormal discharges; three points: three spike foci with <50% abnormal channels and absence of hypsarrhythmia; two points: <3 spike foci without hypsarrhythmia. Post-treatment EEG improvement was defined by either: (1) Baseline scores 5 decreasing to ≤3 post-therapy; (2) Baseline score 3 decreasing to ≤2 post-therapy. Quantitative comparisons were conducted between hypsarrhythmia and non-hypsarrhythmia groups pre- and post-ACTH treatment.

### 2.6 Serum IGF - 1 and IGFBP3 detection

The levels of IGF-1 and IGFBP-3 in peripheral venous blood were measured by chemiluminescence assay in infants with infantile spasms before and after ACTH treatment. All analyses were performed in the clinical laboratory of the research center. IGF-1 was measured using an insulin-like growth factor-I assay kit (SIEMENS), and IGFBP-3 was measured using an insulin-like growth factor-binding protein-3 assay kit (SIEMENS). The analyses were performed on the IMMULITE@2000XPi immunoassay system. All procedures were performed according to the manufacturer’s instructions.

### 2.7 Statistical analysis

Statistical analysis was conducted using SPSS (Version 26). Data were expressed as mean ± SD (range). Normally distributed continuous data were compared with independent t-tests and non-normally distributed data were analyzed using Mann-Whitney U tests. Categorical variables were presented as percentages and compared via chi-square tests. Spearman’s rank correlation coefficient evaluated associations between biomarkers, EEG patterns, and short-term treatment responses. P value < 0.05 indicated statistical significance.

## 3 Results

### 3.1 Clinical characteristic

**FIGURE 1:**
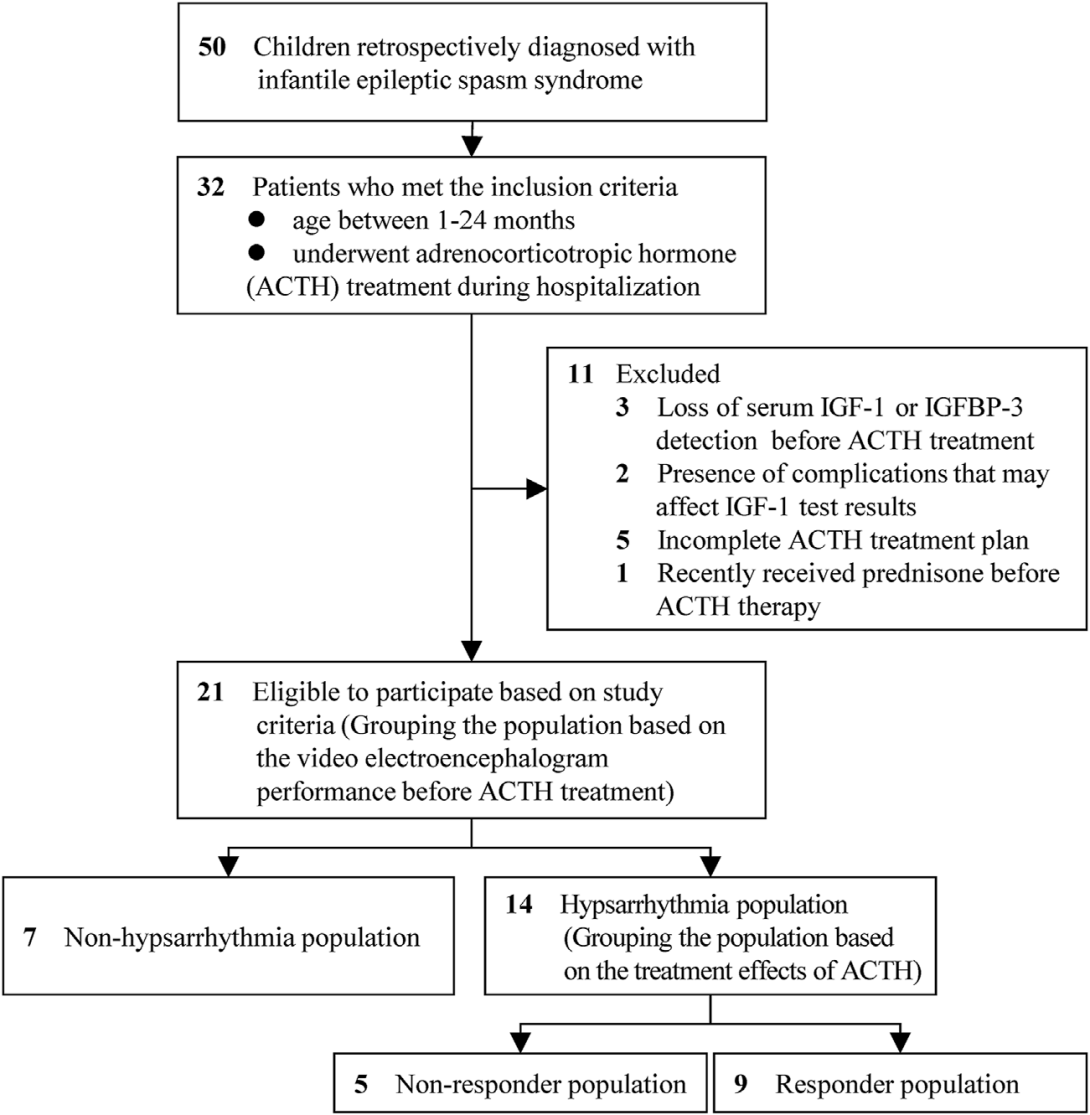
Study flow chart. Abbreviations: IGF-1, Insulin-like Growth Factor 1; IGFBP-3, Insulin-like Growth Factor Binding Protein 3.

As shown in Figure 1, a total of 21 patients diagnosed with IESS and meeting the inclusion/exclusion criteria were enrolled, comprising 14 males and 7 females. Based on the presence of hypsarrhythmia on pre-treatment VEEG, the cohort was divided into a hypsarrhythmia group (14 cases) and a non-hypsarrhythmia group (7 cases). No statistically significant differences were observed between the two groups in terms of gender, age, height, weight, birth history, cranial MRI findings, age of onset, or medication status (Table 1).

**TABLE 1:** Baseline clinical characteristic.

Index	Non-hypsarrhythmia population (n = 7)	Hypsarrhythmia population (n = 14)	*P* value
Sex, male/female	5/2	9/5	1.000
Age, (range), months	9.02 ± 7.00 (2.97–21.90)	6.41 ± 2.40 (1.60–9.77)	0.370
Height, (range), cm	69.93 ± 8.58 (61.00–85.00)	67.46 ± 5.34 (57.00–76.00)	0.425
Weight, (range), kg	9.01 ± 3.05 (5.00–14.50)	7.95 ± 1.62 (4.80–11.80)	0.302
Onset of spasms at <3 months age	2 (28.57%)	3 (21.43%)	1.000
*T*_ot_, (range), months	3.02 ± 4.17 (0.37–12.27)	1.53 ± 2.26 (0.27–8.90)	0.355
Birtd history (%)			
Preterm infant at <37 weeks	2 (28.57%)	3 (21.43%)	1.000
Birth weight <2,500 g	2 (28.57%)	1 (7.14%)	0.247
Breastfeeding	5 (71.43%)	11 (78.58%)	1.000
Neonatal brain injury history	0 (0)	3 (21.43%)	0.521
Abnormal brain MRI	3 (42.86%)	8 (57.14%)	0.659
Seizure frequency ≤5/day (%)	3 (42.86%)	9 (62.29%)	0.397
Medication history before treatment			
Vigabatrin	2 (28.57%)	7 (50.00%)	0.642
Topiramate	1 (14.29%)	3 (21.43%)	1.000
Valproate	0 (0)	2 (14.29%)	0.533

The data in the table were obtained before the patient received adrenocorticotropic hormone treatment. Results were presented as Mean ± SD. Abbreviations: *T*_ot_, time from spasms onset to adrenocorticotropic hormone treatment; MRI, magnetic resonance imaging.

### 3.2 Comparison of the levels of serum IGF-1, IGFBP-3 and its ratio between the non-hypsarrhythmia and hypsarrhythmia groups

**FIGURE 2:**
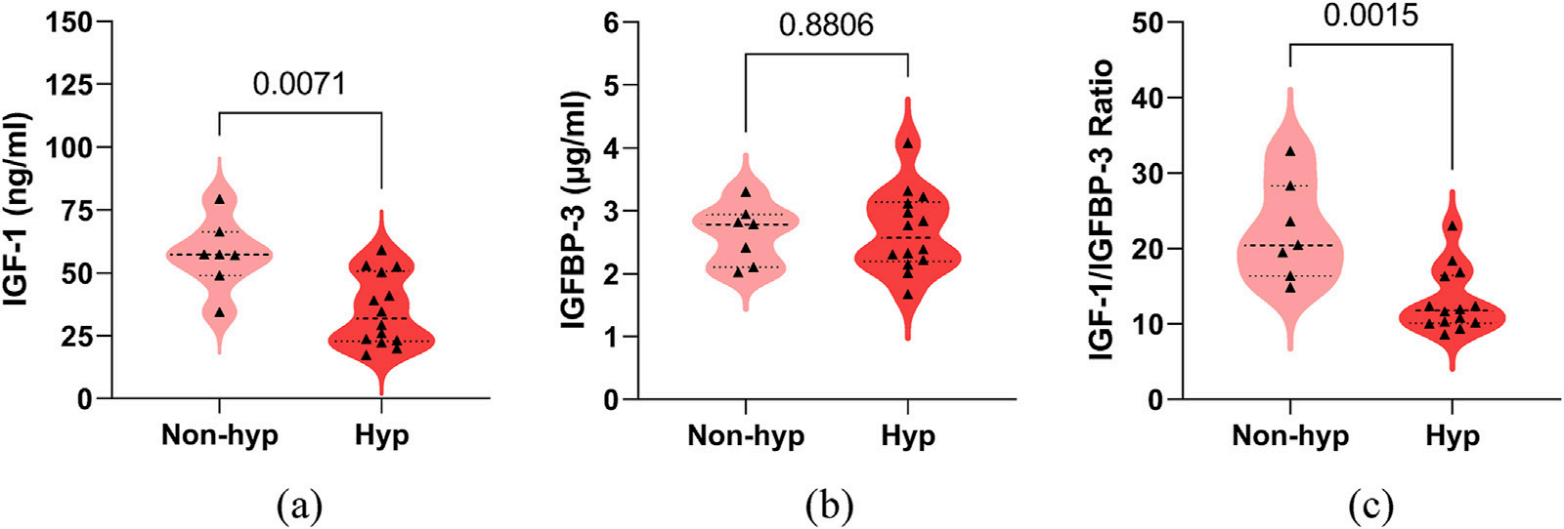
Comparison of serum IGF-1, IGFBP-3, their ratio between the non-hypsarrhythmia (Non-hyp) and hypsarrhythmia (Hyp) population. **(a)** IGF-1; **(b)** IGFBP-3; **(c)** IGF-1 to IGFBP-3 Ratio. The P-values for the comparison between the two groups are listed on the horizontal line. Abbreviations: IGF-1, Insulin-like Growth Factor 1; IGFBP-3, Insulin-like Growth Factor Binding Protein 3.

**FIGURE 3:**
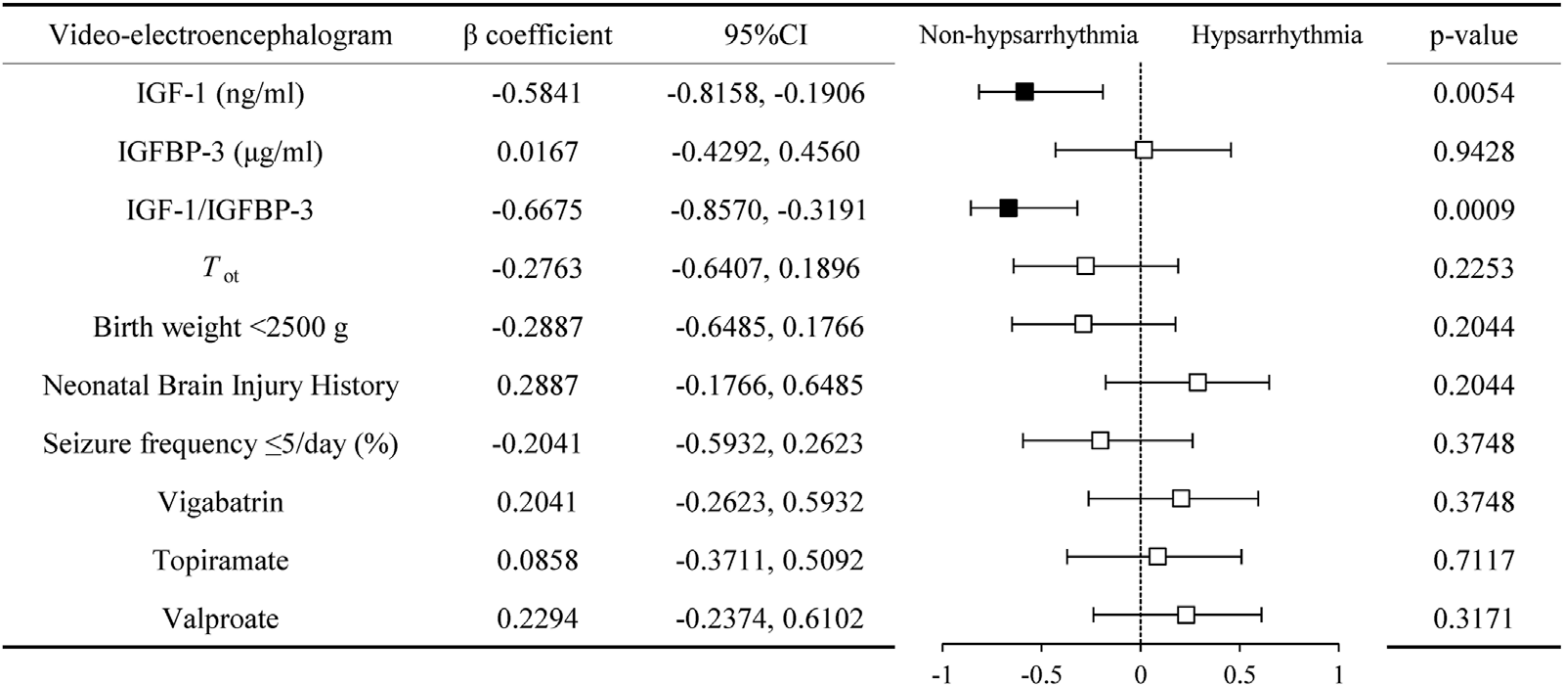
Correlation of serum IGF-1, IGFBP-3, and their ratio with video-electroencephalogram manifestations. Abbreviations: IGF-1, Insulin-like Growth Factor 1; IGFBP-3, Insulin-like Growth Factor Binding Protein 3; *T*_ot_, time from spasms onset to adrenocorticotropic hormone treatment.

We compared the serum IGF-1 and IGFBP-3 levels, as well as the IGF-1/IGFBP-3 ratio, between the hypsarrhythmia and non-hypsarrhythmia groups before ACTH treatment. The results demonstrated that the hypsarrhythmia population had significantly lower serum IGF-1 levels (34.95 ± 14.09, [range 17.20–59.00] vs. 57.15 ± 13.88, [range 34.40–79.30] ng/mL, P = 0.0071) and a significantly reduced IGF-1/IGFBP-3 ratio (12.98 ± 4.11, [range 8.56–22.96] vs. 22.27 ± 6.48, [range 14.85–32.90], P = 0.0015) compared to the non-hypsarrhythmia population (Figure 2). Spearman’s correlation analysis revealed a negative correlation between hypsarrhythmia and serum IGF-1 levels (−0.5841 [95% CI, −0.8158 to −0.1906], P = 0.0054), IGF-1/IGFBP-3 ratio (−0.6675 [95% CI, −0.8570 to −0.3191], P = 0.0009), indicating that lower IGF-1 levels and IGF-1/IGFBP-3 ratios were associated with a higher likelihood of hypsarrhythmia. Additionally, correlation analyses were performed for potential confounders affecting IGF-1 levels, including the interval from seizure onset to ACTH initiation (−0.2763 [95% CI, −0.6407 to 0.1896], P = 0.2253), birth weight (−0.2887 [95% CI, −0.6485 to 0.1766], P = 0.2044), seizure frequency (−0.2041 [95% CI, −0.5932 to 0.2623], P = 0.3748), and medication history (0.2041 [95% CI, −0.2623 to 0.5932]/0.0858 [95% CI, −0.3711 to 0.5092]/0.2294 [95% CI, −0.2374 to 0.6102], P = 0.3748/0.7117/0.3171) (Figure 3); however, no statistically significant associations were observed.

### 3.3 Comparison of the levels of serum IGF-1, IGFBP-3 and its ratio between non-responder and responder groups in IESS patients with hypsarrhythmia

In the hypsarrhythmia subgroup, we compared clinical characteristics between treatment responders (n = 9) and non-responders (n = 5), with no significant intergroup differences observed (Supplementary Table S1). To identify potential biomarkers for predicting therapeutic outcomes, we analyzed associations between pre-ACTH serum IGF-1, IGFBP-3, and IGF-1/IGFBP-3 ratio levels and short-term treatment response.

**FIGURE 4:**
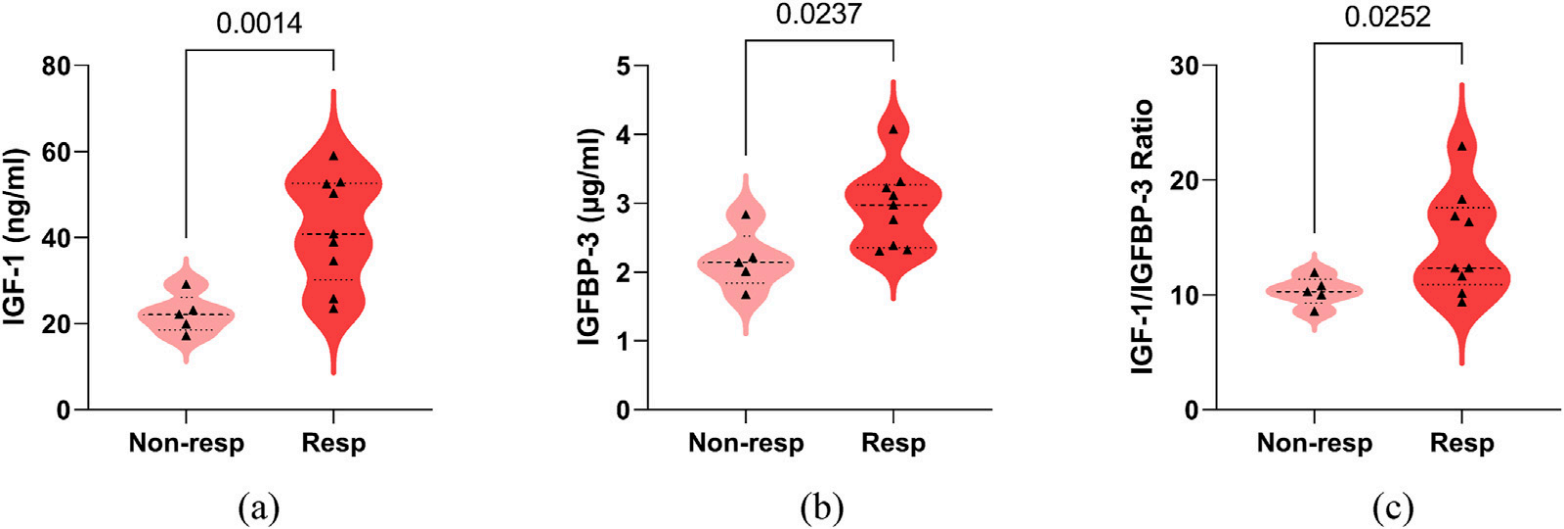
Comparison of serum IGF-1, IGFBP-3, their ratio between adrenocorticotropic hormone treatment non-responder (Non-resp) and responder (Resp) in infantile spasms children with hypsarrhythmia. **(a)** IGF-1; **(b)** IGFBP-3; **(c)** IGF-1 to IGFBP-3 Ratio. The P-values for the comparison between the two groups are listed on the horizontal line. Abbreviations: IGF-1, Insulin-like Growth Factor 1; IGFBP-3, Insulin-like Growth Factor Binding Protein 3.

**FIGURE 5:**
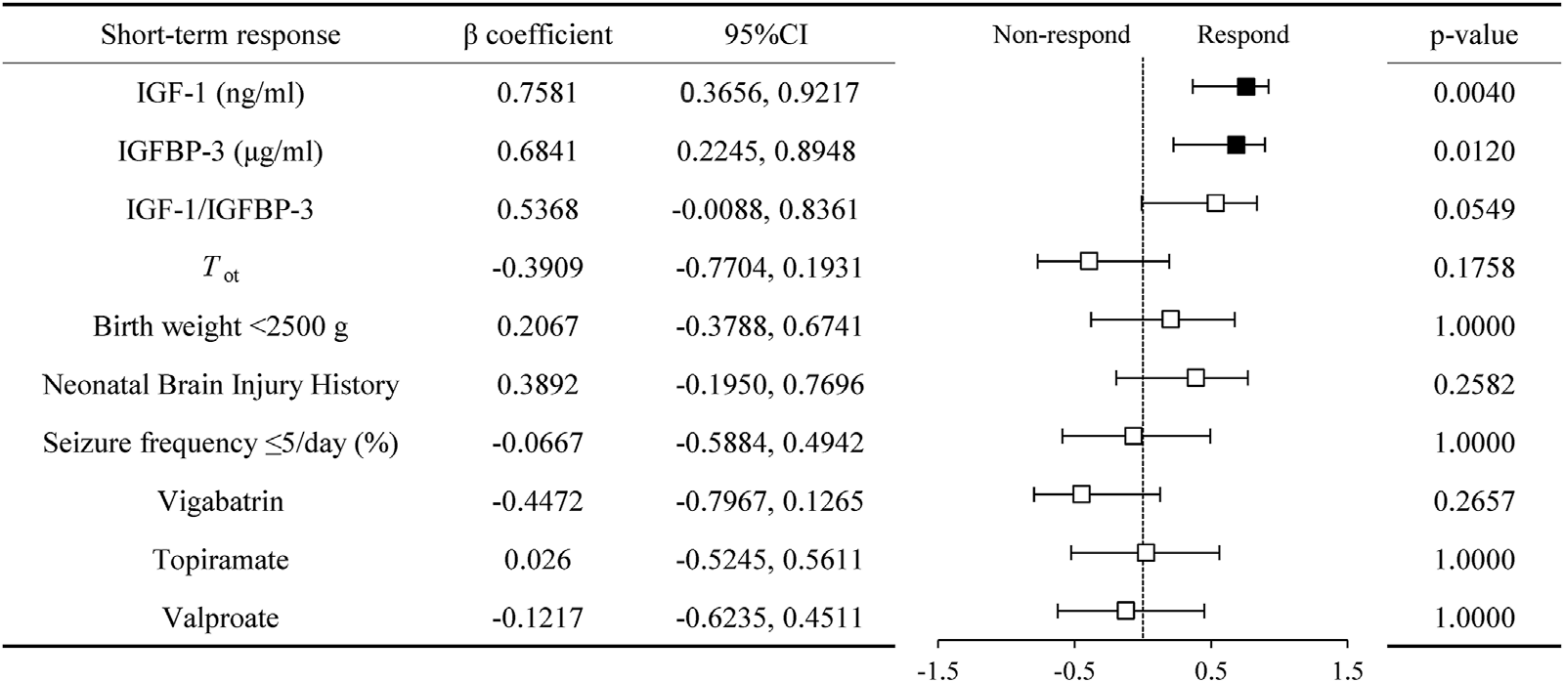
Correlation of serum IGF-1, IGFBP-3, and their ratio with short term respond in infantile spasms children with hypsarrhythmia. Abbreviations: IGF-1, Insulin-like Growth Factor 1; IGFBP-3, Insulin-like Growth Factor Binding Protein 3; *T*_ot_, time from spasms onset to adrenocorticotropic hormone treatment.

Responders demonstrated significantly higher baseline serum IGF-1 (42.00 ± 12.51, [range 23.50–59.00] vs. 22.28 ± 4.44, [range 17.20–29.10] ng/mL, P = 0.0014), IGFBP-3 (2.94 ± 0.58, [range 2.30–4.07] vs. 2.17 ± 0.42, [range 1.67–2.83] μg/mL, P = 0.0237), and IGF-1/IGFBP-3 ratios (14.47 ± 4.45, [range 9.35–22.96] vs. 10.31 ± 1.22, [range 8.56–11.92], P = 0.0252) compared to non-responders (Figure 4). Spearman’s correlation analysis revealed positive associations between treatment response and serum IGF-1 (0.7581 [95% CI, 0.3656 to 0.9217], P = 0.0040), IGFBP-3 (0.6841 [95% CI, 0.2245 to 0.8948], P = 0.0120), and their ratio (0.5368 [95% CI, −0.0088 to 0.8361], P = 0.0549) (Figure 5). No significant confounding effects were observed for covariates such as age, sex, or seizure onset age (P > 0.10).

### 3.4 Changes in serum IGF-1, IGFBP-3, its ratio before and after ACTH treatment in IESS patients with hypsarrhythmia

**FIGURE 6:**
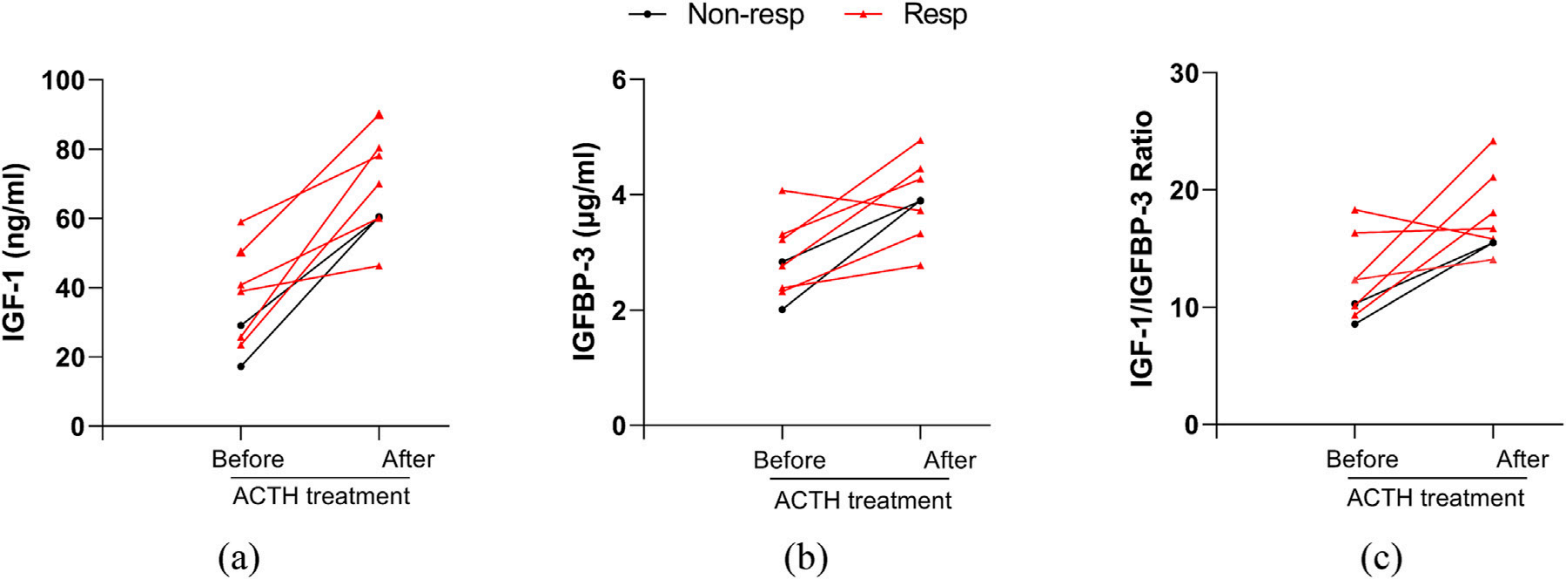
Changes in serum IGF-1, IGFBP-3, their ratio before and after ACTH treatment in patients with hypsarrhythmia. **(a)** IGF-1; **(b)** IGFBP-3; **(c)** IGF-1 to IGFBP-3 Ratio. Abbreviations: Non-resp, non-responder; Resp, responder; IGF-1, Insulin-like Growth Factor 1; IGFBP-3, Insulin-like Growth Factor Binding Protein 3.

We analyzed serum IGF-1, IGFBP-3 levels, and IGF-1/IGFBP-3 ratios in the hypsarrhythmia subgroup before and after ACTH therapy. Due to limited post-treatment sampling (six responders, two non-responders), results demonstrated that ACTH universally elevated serum IGF-1 and IGFBP-3 levels regardless of baseline values. Responders exhibited a higher magnitude of IGF-1(39.70 ± 13.71, [range 23.50–59.00] vs. 70.82 ± 15.68, [range 46.30–90.00] ng/mL) increase compared to non-responders (17.20; 29.10 vs. 60.20; 60.50 ng/mL) (Figure 6), suggesting that dynamic monitoring of these biomarkers may guide therapeutic adjustments. Current findings require validation in larger cohorts to confirm prognostic utility.

## 4 Discussion

This study examined how serum IGF-1 levels, IGFBP-3 levels, and their ratio relate to EEG hypsarrhythmia and ACTH therapy response in IESS patients, demonstrating the potential clinical value of peripheral IGF-1.

Hypsarrhythmia correlates strongly with disease progression, prognosis, and treatment response. A retrospective study of 48 patients demonstrated that prolonged hypsarrhythmia (>3 weeks) was associated with cognitive impairment (Primec et al., 2006). However, current research lacks a thorough exploration of the relationship between serum IGF-1 levels and IESS-related EEG characteristics. Preclinical trials employing IGF-1-derived tripeptides have demonstrated efficacy in reducing spasms and hypsarrhythmia through IGF-1 pathway activation (Ballester-Rosado et al., 2025). Our study links serum IGF-1 levels to EEG features, demonstrating that both lower IGF-1 levels and reduced IGF-1/IGFBP-3 ratios correlate with greater EEG background disorganization. Hypsarrhythmia inversely correlated with both serum IGF-1 levels and IGF-1/IGFBP-3 ratios, consistent with previous findings, suggesting that decreased ratios may indicate reduced bioactive IGF-1, thereby worsening hypsarrhythmia. Emerging research proposes peripheral blood biomarkers, including IGF-1, for epilepsy classification, providing new diagnostic approaches (Razia et al., 2024). These findings support IGF-1 as a biomarker for early diagnosis and severity assessment in IESS.

Clinically, serum IGF-1 levels show association with factors such as birth weight. Research on healthy infants has revealed an inverse correlation between serum IGF-1 concentration at 3 months of age (Chellakooty et al., 2006). While we observed a similar inverse relationship in our cohort, the association was not statistically significant, likely due to our smaller sample size. By implementing strict inclusion/exclusion criteria to account for potential confounders such as diabetes, infection, and hypothyroidism, our findings suggest a potential independent negative association between serum IGF-1 levels and hypsarrhythmia that warrants further investigation.

Serum IGF-1 levels may serve as a predictor of therapeutic response in IESS patients exhibiting hypsarrhythmia on EEG. In surgically resected brain tissues from IESS patients, immunohistochemical analysis revealed IGF-1 expression patterns consistent with those observed in mouse models (Dang, 2023). Clinical studies have linked higher CSF IGF-1 levels to favorable ACTH responses and improved cognitive outcomes (Riikonen et al., 2010).

In this study, among IESS patients in the hypsarrhythmia subgroup, those with higher baseline serum levels of IGF-1, IGFBP-3, and their ratio prior to treatment demonstrated a significantly better response to ACTH therapy. Correlational analysis further confirmed a positive association between these serum biomarkers and therapeutic efficacy. Among the 11 patients with MRI abnormalities (including periventricular leukomalacia or corpus callosum dysgenesis), IGF-1 levels (35.96 ± 15.53, [range 17.20–66.20] ng/mL) were lower than those in patients with non-structural etiologies (49.38 ± 16.46, [range 19.90–79.30] ng/mL), though this difference did not reach statistical significance (p = 0.099). No significant correlation was observed between MRI structural abnormalities and short-term treatment response. Patients with higher IGF-1 levels exhibited an increased probability for positive response to ACTH, providing preliminary evidence for future use of IGF-1 as a predictive biomarker. This finding warrants further validation through larger-scale studies employing larger sample sizes to clarify its clinical relevance.

Furthermore, IGF-1 and IGFBP-3 are recognized to play critical roles in modulating blood-brain barrier permeability and neuroinflammatory responses. Studies demonstrate IGF-1’s neuroprotective effects via multiple signaling pathways, including PI3K/Akt/mTOR and Ras/ERK. Both clinical trials and animal studies show that externally administered IGF-1 can reduce neural damage while improving motor and cognitive functions, underscoring its therapeutic potential for neurodegenerative disorders such as epilepsy (Ge et al., 2022; Bhalla et al., 2022). In this study, the concomitant elevation of IGFBP-3 levels in the responder group further supports the proposed mechanism: IGFBP-3 may synergistically enhance anti-inflammatory and neurorestorative effects by stabilizing IGF-1 and prolonging its half-life. This discovery provides the theoretical groundwork for developing an integrated predictive model based on the IGF-1/IGFBP-3 ratio.

The modulation of IGF-1 may influence both the frequency and severity of epileptic seizures, thus providing a rationale for developing therapeutic strategies. Dynamic monitoring of IGF-1 levels and implementing targeted interventions could potentially improve clinical prognosis in IESS patients. Preclinical animal studies demonstrate the therapeutic potential of an IGF-1-derived tripeptide treatment, which shows significant anticonvulsant effects and suppresses hypsarrhythmia on EEG (Ballester-Rosado et al., 2025).

In this study, longitudinal monitoring of IGF-1 levels before and after ACTH therapy was performed in a subset of patients. Post-treatment analysis revealed significantly elevated IGF-1 and IGFBP-3 levels in the entire cohort, with the responder subgroup exhibiting even higher IGF-1 concentrations. These findings suggest that dynamic monitoring of IGF-1 may serve as a novel biomarker to guide clinical decision-making and refine therapeutic strategies. While IGF-1 demonstrates substantial predictive and therapeutic potential, its clinical translation faces challenges, including absence of standardized protocols for biomarker quantification and limited understanding of its mechanistic roles across heterogeneous diseases (Huang et al., 2024).

This study is the first to establish correlations between serum IGF-1 levels, EEG characteristics of IESS, and short-term ACTH therapeutic response. These findings suggest IGF-1 as a potential biomarker for both diagnostic stratification and therapeutic personalization. Specifically, the integration of baseline IGF-1 quantification with EEG patterns may improve diagnostic accuracy, objectively evaluate disease severity, and guide tailored therapeutic regimens. For instance, patients exhibiting low baseline IGF-1 levels combined with hypsarrhythmia EEG patterns might benefit from early intensive interventions, such as ACTH dose escalation or combination therapy with vigabatrin. Furthermore, these findings enable future IGF-1-based therapy protocols by defining pharmacodynamic parameters for optimal dosing.

### 4.1 Study limitations and future directions

1) The small non-hypsarrhythmia subgroup (n = 7) limits the study’s generalizability; 2) The lack of longitudinal IGF-1 profiling prevents accurate correlation between peak IGF-1 levels and spasm control timing; 3) Uncontrolled confounders including variations in birth weight and differences in treatment initiation timing; 4) Parental reports of spasm resolution may contain reporting bias. Future multicenter prospective studies should incorporate both CSF and peripheral blood IGF-1 assays, combined with molecular pathway profiling, to fully elucidate IGF-1’s epileptogenic mechanisms.

## Data Availability

The raw data supporting the conclusion of this article will be made available by the authors, without undue reservation.
