# Supplementary material for: Association of serum insulin-like growth factor-1 and adrenocorticotropic hormone therapeutic response in patients with infantile epileptic spasms syndrome
Source: Front Pharmacol. 2025 Apr 30;16:1599641. doi: 10.3389/fphar.2025.1599641 (PMC12075540; doi:10.3389/fphar.2025.1599641)
Supplement: Supplementary file 1 [file Table1.docx]

**Table S1**. Clinical characteristic of the subjects in Hypsarrhythmia population before adrenocorticotropic hormone treatment.

| Index | Non-responder  population  (n=5) | Responder  population  (n=9) | *P* value |
| --- | --- | --- | --- |
| Sex, male/female | 4/1 | 5/4 | 0.580 |
| Age, (range), months | 7.23±3.32  (1.60-9.77) | 6.00±1.79  (3.30-9.33) | 0.362 |
| Height, (range), cm | 67.20±7.79  (57.00-76.00) | 67.61±3.98  (61.00-73.00) | 0.897 |
| Weight, (range), kg | 7.78±2.55  (4.80-11.80) | 8.04±1.00  (6.40-9.30) | 0.788 |
| Onset of spasms at＜3 months age | 1 (20.00%) | 2 (22.22%) | 1.000 |
| *T*_ot_, (range), months^†^ | 1.44±1.14  (0.60-3.40) | 1.59±2.77  (0.27-8.90) | 0.176 |
| Birth history (%) |  |  |  |
| Preterm infant at＜37 weeks | 1 (20.00%) | 2 (22.22%) | 1.000 |
| Birth weight <2500 g | 0 (0) | 1 (11.11%) | 1.000 |
| Breastfeeding | 5 (100%) | 6 (66.67%) | 0.258 |
| Neonatal Brain Injury History | 0 (0) | 3 (33.33%) | 0.258 |
| Abnormal brain MRI | 4 (80.00%) | 4 (44.44%) | 0.301 |
| Seizure frequency ≤5/day (%) | 3 (60.00%) | 6 (66.67%) | 1.000 |
| Medication history before treatment |  |  |  |
| Vigabatrin | 4 (80.00%) | 3 (33.33%) | 0.266 |
| Topiramate | 1 (20.00%) | 2 (22.22%) | 1.000 |
| Valproate | 1 (20.00%) | 1 (11.11%) | 1.000 |

Results were presented as Mean±SD.

Abbreviations: *T*_ot_, time from spasms onset to adrenocorticotropic hormone treatment, MRI, magnetic resonance imaging.

**Table S2**. Duration and dosage of ACTH treatment for the subjects

| Case  number | Initial medication time | Initial dosage  (IU) | First adjustment time | First adjustment dosage (IU) | Second  adjustment time | Second  adjustment dose (IU) | Third  adjustment time | Third  adjustment dose (IU) | Adjustment  Number | Final dosage (IU) | Total duration  (days) |
| --- | --- | --- | --- | --- | --- | --- | --- | --- | --- | --- | --- |
| 1 | 2021/7/25 | 12.5 | 2021/7/30 | 25 | / | / | / | / | 1 | 25 | 14 |
| 2 | 2021/12/8 | 25 | / | / | / | / | / | / | 0 | 25 | 14 |
| 3 | 2022/1/17 | 12.5 | 2022/1/21 | 25 | / | / | / | / | 1 | 25 | 14 |
| 4 | 2022/1/21 | 25 | / | / | / | / | / | / | 0 | 25 | 14 |
| 5 | 2022/1/22 | 25 | / | / |  | / | / | / | 0 | 25 | 14 |
| 6 | 2022/2/17 | 25 | 2022/2/25 | 50 | / | / | / | / | 1 | 50 | 14 |
| 7 | 2022/8/9 | 25 | 2022.08.15 | 50 | / | / | / | / | 1 | 50 | 14 |
| 8 | 2022/8/11 | 12.5 | 2022.8.13 | 25 | 2022.8.19 | 37.5 | 2022.8.20 | 50 | 3 | 50 | 14 |
| 9 | 2022/9/28 | 25 | 2022.10.4 | 50 | / | / | / | / | 1 | 50 | 14 |
| 10 | 2022/11/16 | 25 | 2022.11.21 | 12.5 | / | / | / | / | 1 | 25 | 22 |
| 11 | 2022/12/15 | 25 | / | / | / | / | / | / | 0 | 25 | 7 |
| 12 | 2023/2/25 | 12.5 | 2023/2/28 | 25 | / | / | / | / | 1 | 25 | 14 |
| 13 | 2023/6/11 | 25 | / | / | / | / | / | / | 0 | 25 | 13 |
| 14 | 2023/8/8 | 25 | / | / | / | / | / | / | 0 | 25 | 13 |
| 15 | 2023/9/26 | 25 | / | / | / | / | / | / | 0 | 25 | 13 |
| 16 | 2024/6/24 | 25 | 2024/6/24 | / | / | / | / | / | 0 | 25 | 14 |
| 17 | 2024/7/4 | 25 | 2024/7/4 | / | / | / | / | / | 0 | 25 | 14 |
| 18 | 2024/8/27 | 12.5 | 2024/8/29 | 25 | 2024/9/3 | 50（25 bid） |  |  | 2 | 50 | 14 |
| 19 | 2024/11/16 | 25 | 2024/11/25 | 50（25 bid） |  |  |  |  | 1 | 50 | 14 |
| 20 | 2024/11/16 | 25 | 2024/11/25 | 50（25 bid） |  |  |  |  | 1 | 50 | 14 |
| 21 | 2023/4/21 | 25 | / | / | / | / | / | / | 0 | 25 | 14 |

**Table S3.** Assessment of Epileptic Spasms (ES) in Subjects Before and After ACTH Treatment

| Case  number | Number of ES per day before treatment | Number of ES per day after treatment | Epileptic Spasms assessment  (0. ES-unceased; 1. ES-free) |
| --- | --- | --- | --- |
| 1 | 7~8 | 4~5 | 0 |
| 2 | 4~5 | 0 | 1 |
| 3 | 2 | 0 | 1 |
| 4 | 4~5 | 4~5 | 0 |
| 5 | 5~6 | 0 | 1 |
| 6 | 5~6 | 4 | 0 |
| 7 | 10+ | 0 | 1 |
| 8 | 10+ | 0 | 1 |
| 9 | 3~4 | 0 | 1 |
| 10 | 2~3 | 0 | 1 |
| 11 | 3~5 | 0 | 1 |
| 12 | 5~6 | 5~6 | 0 |
| 13 | 4~5 | 4~5 | 0 |
| 14 | 5~6 | 0 | 1 |
| 15 | 5 | 0 | 1 |
| 16 | 10+ | 0 | 1 |
| 17 | 2~3 | 0 | 1 |
| 18 | 2~4 | 1 | 0 |
| 19 | 3~4 | 0 | 1 |
| 20 | 3 | 1 | 0 |
| 21 | ＞5 | 0 | 1 |

**Table S4.** Assessment of VEEG improvement in Subjects Before and After ACTH Treatment

| Case number | VEEG before treatment | VEEG after treatment | VEEG improvement assessment（0.No; 1.Yes，） |
| --- | --- | --- | --- |
| 1 | Epileptic discharge in the right hemisphere，spasms | Spasms | 0 |
| 2 | Epileptic discharge | Normal | 1 |
| 3 | Hypsarrhythmia | Epileptic discharge，non-hypsarrhythmia | 1 |
| 4 | Hypsarrhythmia | Spasms | 0 |
| 5 | Hypsarrhythmia，spasms | Normal | 1 |
| 6 | Epileptic discharge，spasms | Normal | 1 |
| 7 | Hypsarrhythmia，spasms | Non-hypsarrhythmia | 1 |
| 8 | Hypsarrhythmia | Hypsarrhythmia | 0 |
| 9 | Hypsarrhythmia，spasms | Epileptic discharge，non-hypsarrhythmia | 1 |
| 10 | Hypsarrhythmia，spasms | Epileptic discharge，non-hypsarrhythmia | 1 |
| 11 | Hypsarrhythmia，spasms | Epileptic discharge，non-hypsarrhythmia | 1 |
| 12 | Hypsarrhythmia | Hypsarrhythmia | 0 |
| 13 | Hypsarrhythmia，spasms | Hypsarrhythmia，spasms | 0 |
| 14 | burst suppression，spasms | Epileptic discharge，non-hypsarrhythmia | 1 |
| 15 | Epileptic discharge，spasms | Epileptic discharge，non-hypsarrhythmia | 1 |
| 16 | Hypsarrhythmia，spasms | Epileptic discharge，non-hypsarrhythmia | 1 |
| 17 | Hypsarrhythmia，spasms | Epileptic discharge，non-hypsarrhythmia | 1 |
| 18 | Hypsarrhythmia，spasms | Spasms | 0 |
| 19 | Hypsarrhythmia，spasms | Epileptic discharge，non-hypsarrhythmia | 1 |
| 20 | Epileptic discharge | Epileptic discharge | 0 |
| 21 | Epileptic discharge | Epileptic discharge | 0 |

**Table S5** Changes in serum IGF-1, IGFBP-3, their ratio before and after ACTH treatment in patients with hypsarrhythmia.

| Case  number | Before ACTH treatment | | | |  | After ACTH treatment | | | |
| --- | --- | --- | --- | --- | --- | --- | --- | --- | --- |
|  | Time | IGF-1  (ng/mL) | IGFBP-3  (μg/mL) | IGF-1/IGFBP-3 |  | Time | IGF-1  (ng/mL) | IGFBP-3  (μg/mL) | IGF-1/IGFBP-3 |
| 9 | 28-Sep-22 | 23.5 | 2.32 | 10.13 |  | 10-Oct-22 | 70 | 3.32 | 21.08 |
| 10 | 15-Nov-22 | 40.8 | 3.31 | 12.33 |  | 01-Dec-22 | 60.1 | 4.27 | 14.07 |
| 11 | 14-Dec-22 | 38.9 | 2.38 | 16.34 |  | 22-Dec-22 | 46.3 | 2.77 | 16.71 |
| 13 | 04-Jun-23 | 17.2 | 2.01 | 8.56 |  | 24-Jun-23 | 60.5 | 3.9 | 15.51 |
| 16 | 25-Jun-24 | 25.8 | 2.76 | 9.35 |  | 08-Jul-24 | 80.4 | 4.45 | 18.07 |
| 17 | 28-Jun-24 | 59 | 3.22 | 18.32 |  | 17-Jul-24 | 78.1 | 4.94 | 15.81 |
| 18 | 26-Aug-24 | 29.1 | 2.83 | 10.28 |  | 11-Sep-24 | 60.2 | 3.89 | 15.48 |
| 19 | 14-Nov-24 | 50.2 | 4.07 | 12.33 |  | 29-Nov-24 | 90 | 3.72 | 24.19 |

**Table S6.** Age, height, and weight data of the participating subjects

| Case  number | Age  (months) | Height  (cm) | Weight  (kg) |
| --- | --- | --- | --- |
| 1 | 2.97 | 61 | 5 |
| 2 | 8.73 | 65 | 10 |
| 3 | 7.77 | 70 | 8.3 |
| 4 | 7.5 | 64 | 8 |
| 5 | 9.33 | 70 | 8.54 |
| 6 | 4.73 | 72 | 8.5 |
| 7 | 6.63 | 70 | 8.5 |
| 8 | 7.67 | 74 | 7.3 |
| 9 | 4.63 | 61 | 7.5 |
| 10 | 5.9 | 70 | 8 |
| 11 | 5.17 | 63 | 9.3 |
| 12 | 1.6 | 57 | 4.8 |
| 13 | 9.77 | 65 | 7 |
| 14 | 3.27 | 61 | 6.3 |
| 15 | 6.73 | 70.5 | 8.8 |
| 16 | 4.97 | 67 | 6.7 |
| 17 | 3.3 | 64.5 | 6.4 |
| 18 | 9.6 | 76 | 11.8 |
| 19 | 5.9 | 73 | 9.1 |
| 20 | 14.83 | 75 | 10 |
| 21 | 21.9 | 85 | 14.5 |
